# Facing Off with Unfair Others: Introducing Proxemic Imaging as an Implicit Measure of Approach and Avoidance during Social Interaction

**DOI:** 10.1371/journal.pone.0117532

**Published:** 2015-02-12

**Authors:** Cade McCall, Tania Singer

**Affiliations:** Department of Social Neuroscience, Max Planck Institute for Human Cognitive and Brain Sciences, Leipzig, Germany; Max Planck Institute for Human Cognitive and Brain Sciences, GERMANY

## Abstract

Nonverbal behavior expresses many of the dynamics underlying face-to-face social interactions, implicitly revealing one’s attitudes, emotions, and social motives. Although research has often described nonverbal behavior as approach versus avoidant (i.e., through the study of proxemics), psychological responses to many social contexts are a mix of these two. Fairness violations are an ideal example, eliciting strong avoidance-related responses such as negative attitudes, as well as strong approach-related responses such as anger and retaliation. As such, nonverbal behavior toward unfair others is difficult to predict in discrete approach versus avoidance terms. Here we address this problem using proxemic imaging, a new method which creates frequency images of dyadic space by combining motion capture data of interpersonal distance and gaze to provide an objective but nuanced analysis of social interactions. Participants first played an economic game with fair and unfair players and then encountered them in an unrelated task in a virtual environment. Afterwards, they could monetarily punish the other players. Proxemic images of the interactions demonstrate that, overall, participants kept the fair player closer. However, participants who actively punished the unfair players were more likely to stand directly in front of those players and even to turn their backs on them. Together these patterns illustrate that fairness violations influence nonverbal behavior in ways that further predict differences in more overt behavior (i.e., financial punishment). Moreover, they demonstrate that proxemic imaging can detect subtle combinations of approach and avoidance behavior during face-to-face social interactions.

## Introduction

### Responses to unfair others

No one likes to be treated unfairly. Research using economic games has repeatedly shown that unfair behavior provokes a range of negative responses. At the level of attitudes, unfair others are perceived as less likeable, less agreeable, and even less attractive than fair others [1,2]. At the level of subjective emotional experience, people who have been treated unfairly report feeling disgust, anger, and even sadness [3,4]. Many people are so motivated to retaliate against unfair behavior that they will act against rational self-interest and spend their own money to monetarily punish unfair players in economic exchange games [5], even when they are merely third-party observers of the unfair behavior [6].

Our negative reactions to unfairness are not surprising given how central fairness is to cooperation and successful group living in general [7,8]. Whether on the Savanna or the office, successful cooperation depends on group members doing their fair share. Unfair others are not trustworthy, cannot be relied upon to reciprocate, and violate group norms (e.g., [9,10]). As such, they are obvious targets for avoidance and/or censure.

Although fairness has been extensively investigated, especially in behavioral economics, most empirical research has focused on overt behavior such as monetary choices. But what happens in everyday life when we encounter unfair others, but don’t have the opportunity to explicitly punish or confront them? Do the powerful negative reactions to unfairness express themselves in other ways, influencing more nonverbal behavior during face-to-face social interactions? And if so, do those relatively subtle nonverbal responses predict more explicit behaviors, i.e. financial punishment, when individuals are given the opportunity to retaliate or censure unfair behavior. To explore these questions, we first consider the nature of nonverbal approach and avoidance during social interactions.

### Proxemics: The study of nonverbal approach and avoidance

Anthropologist Edward C. Hall first coined the term “proxemics” to describe his examinations of the communicative and cultural uses of interpersonal space [11]. Proxemic behaviors include interpersonal distancing, bodily orienting, and the degree to which we gaze at others. Researchers have long argued that these nonverbal channels implicitly express attitudes and motivations during social interactions, and that they reflect the nature of the relationship between two interactants (e.g.,[12,13,14,15]).

At a gross level, proxemic responses can be categorized in terms of the degree to which one approaches or avoids the other person. Not surprisingly, we tend to avoid people whom we evaluate negatively and approach people whom we evaluate positively [15]. This fact is evident in research on implicit prejudice. In early studies, White American participants kept a greater distance between themselves and Black targets [16,17]. More recently, this avoidance of racial outgroup members has also been linked to implicit measures of prejudice [18] and with violence towards those targets [19]. In addition to interpersonal distance, avoidant gaze patterns have also been linked to racial prejudice [19,20]. The relationship between attitudes and proxemics appears to generalize beyond race. For example, participants reporting negative attitudes toward obese people sat further from overweight interaction partners [21].

Importantly, the avoidant proxemic behavior seen in many of these studies correlated with implicit, but not explicit, measures of prejudice [18,19,21]. This pattern suggests that proxemic behavior can be relatively automatic and less conscious than more deliberative and explicit responses such as self-report on questionnaires. As such, proxemic measurement may reveal responses that participants are either unwilling to report or unable to consciously access (e.g., [20,22]).

The avoidant response to negatively valenced targets is also relatively dynamic insofar as people avoid targets in direct response to the target’s behavior. Recent research on close relationships demonstrated that the actions of one’s partner can even elicit avoidance [23]. Participants who had been ignored by their partner during a stressful situation, later kept greater distance from that partner (as compared to participants who had received nonverbal support from their partners).

Together these data lead to the intuitive hypothesis that people avoid disliked others or others who have recently behaved badly. Despite the lack of studies exploring proxemics behavior in the context of fairness, one would therefore expect people to avoid unfair and approach fair others. However, unfairness doesn’t only elicit negative attitudes; it also elicits disgust and anger [3,4]. And although disgust is associated with avoidance, anger is associated with approach [24,25]. Moreover, one of the most interesting features in the response to unfairness is the aggressive desire to punish unfair others, even at a cost to oneself [5]. This aggression is most likely driven by an approach motivation.

As such, extant research provides reason to believe that although unfair behavior might generally elicit avoidant proxemic responses, individuals high in a tendency to punish unfair behavior might actually also show approach patterns toward unfair others. We tested these hypotheses using a new method of proxemic analysis, “proxemic imaging”, designed to provide a relatively nuanced look at approach and avoidance behavior in a dyad.

### Proxemic measurement: A methodological challenge

Measuring proxemic behavior, particularly if it involves a mix of approach and avoidant responses, is a methodological challenge. Over the years, researchers have devised a variety of one-shot measures for proxemics research. These range from simply asking participants to report where they would place themselves with respect to a given target (e.g. [26]), to observing which seat participants choose with respect to a target’s seat (e.g., [27]). More recently, digital motion capture technology has provided researchers with more accurate and near-continuous measurement of a participant’s distance from a target. To reduce this abundance of data into a manageable form, researchers have used the mean or minimum distance from a given target or average gaze direction [18,19,28].

While these measures are precise and objective, one naturally loses information when collapsing an entire interaction into one value. Moreover, interpersonal distance varies significantly with the orientation of the interactants. For example, people tend to come significantly closer to a stranger’s back than front [28]. Depending on the question of interest, this fact may be lost or create noise when measures are aggregated across all sides of a target’s egocentric space. To achieve a better global account of an entire encounter, one might use more subjective methods such as video-coding. But such methods are, of course, highly resource intensive.

Here we present a new method of measuring proxemics that is objective, accounts for an entire interaction, and allows us to look at both interactants’ behaviors simultaneously. Proxemic imaging takes digital tracking data over the length of a social interaction and uses it to create frequency maps of dyadic social space. Because the measurement space is defined by interpersonal distance and the gaze behavior of both people in dyads, proxemic imaging allows us to look not just at an individual’s response but also at the interrelationship between interpersonal distance, one’s own gaze, and the gaze of the other individual. We apply this method to detect differential nonverbal responses during a social interaction with fair versus unfair others and to relate those nonverbal responses to punishment behavior.

## Method

### Participants

Fifty-six (56) participants completed the study (26 women). Given prior research on proxemics (i.e., [23,29]), we sought a sample size of at least 50 and completed running once all scheduled participants had completed the study. One participant was excluded because of technical problems with the motion tracking data. The experimental protocol was in accordance with the principles of the Declaration of Helsinki and was approved by the Ethics Commission of the University of Leipzig (our Institutional Review Board). Before participating, all participants provided informed consent for the study via a written consent form which was approved by the Ethics Commission. Participants received payment for their time as well as a bonus dependent on their earnings in the economic game. They were fully debriefed at the conclusion of the experiment.

### Confederates

Throughout the experiment, participants interacted with two confederates whom they believed to be fellow participants. They met these confederates at the beginning of the experiment while waiting in the lobby. After consenting to participate and receiving a brief introduction, the experimenter told the participants that each would be randomly assigned to a role that they would play during the study (Player A, B, or C). In fact, the participant was always assigned the role of Player B. The experimenter then walked each of the three players to a separate laboratory, explaining that they would interact with each other online for the remainder of the experiment.

### Tasks

The experiment consisted of four main tasks that occurred in the following order: 1) an economic game, 2) an ostensible “memory task” in an immersive virtual environment, 3) an opportunity to financially punish the other players, and 4) a series of post-task questionnaires.

**Fig 1:**
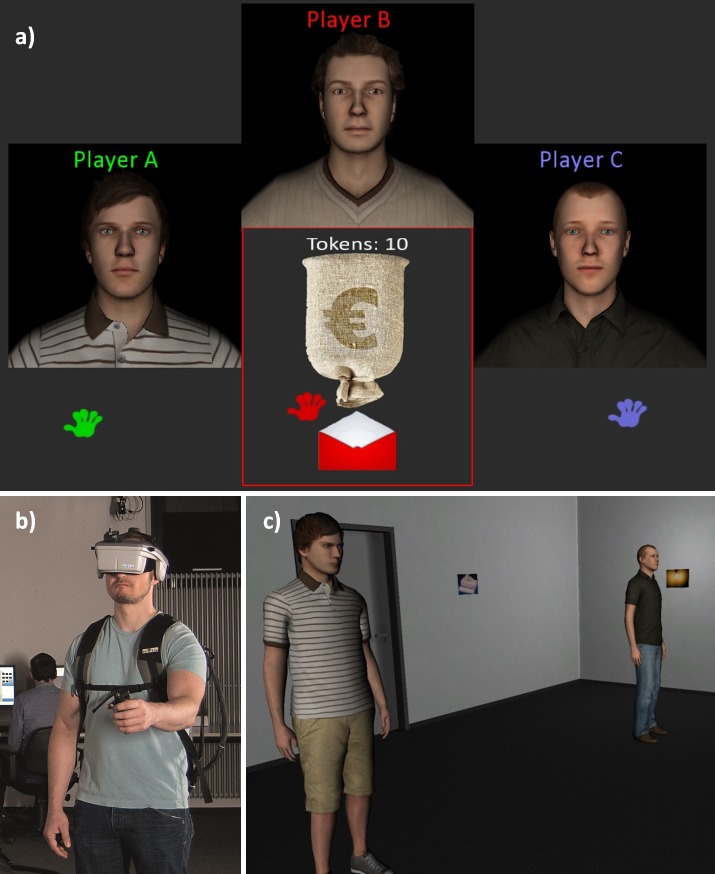
The experiment. Participants completed an economic game (a) with two other players whom they were led to believe were fellow participants represented with avatars. Participants then wore a head mounted display (c) to complete a task in an immersive virtual environment (b) in which they again encountered the other players.

**The Economic Game**. The economic game in this experiment was used to manipulate participants’ perceptions of the other players as fair versus unfair people. This game, played on a desktop computer (Fig. 1A), was a sequential iterated Prisoner’s Dilemma, similar to that used in [2]. Each round of the game involves a first and second player. The first player is given 10 monetary units (MUs) to start. They can then choose to keep those MUs or to transfer them to the second player, at which point the number of MUs is tripled. In the next step, the second player has the option of sending the first player a proportion of those MUs. If they choose to do so, that amount is also tripled. If the first player chooses not to transfer any MUs, the round ends.

Participants were led to believe that as Player B, they would always be the first player in the game and that for each round either Player A or Player C (the confederates) would be randomly chosen to be the second player. Critically, participants made their investment decisions before they knew which of the confederates would be the second player for that round. The participants played the confederates twelve times each. Over the course of these rounds, one player was significantly fairer than the other (sending an average 9 versus 4 MUs to the participant). If participants decided not to invest in the first play, we added a round to the game such that each participant received the same number of exposures to each of the other players. To ensure that participants would associate each confederates’ with that specific confederate, each player was represented in the game with an avatar, ostensibly of their choosing (although the confederate avatars were actually randomly assigned).

**The immersive virtual environment**. The memory task took place in an immersive virtual environment (i.e. “virtual reality”; Fig. 1C). To experience the environment, participants wore a stereoscopic head-mounted display (an NVIS nVisor SX60; Fig. 1B). Head position was tracked using a Worldviz PPT-H tracking system and orientation was tracked with an Intersense InertiaCube 3. The tracking data were used to render the viewpoint in the virtual world such that participants could turn their heads and walk through the world as naturalistically as they would move through the physical world. Meanwhile we recorded the position and orientation of the participant’s head with respect to the other two players at approximately 60 Hz.

The virtual world itself was a simple grey room with pictures on each of the four walls. The other two players’ avatars stood in the room. We led participants to believe that the other players were wearing head-mounted displays in their respective labs and that they were being networked into the bodies of their avatars. We also told participants that the confederates were in labs with less physical space so that although the participant could walk around their virtual world, confederates would need to remain standing.

After a “calibration task” in which the participant and confederate avatars demonstrated their abilities to move and look around (e.g., by nodding at each other), participants completed a task ostensibly designed to study memory. During this task the participant’s goal was to walk around the room and look at pictures. They were told that they would be quizzed after the task to see how well they remembered the pictures. The confederates’ task was ostensibly to look at the pictures in their corner of the room and to watch for green dots appearing on them. The memory task lasted three minutes.

**The punishment round**. During the punishment round, participants were given the opportunity to pay to deduct earnings from each of the other players (e.g., [5]). They were told that for each MU that they paid, the other player would lose 3 MUs.

**Questionnaires**. Finally, participants completed a questionnaire in which they rated the other two players in terms of being fair, likeable, enjoyable, annoying, and attractive [2] on a Likert scale ranging from -3 to +3.

## Results

### Manipulation check

To confirm that the fairness manipulation worked, we ran paired t-tests comparing participants’ subjective responses to the fair versus unfair players. Participants indeed rated the fair player as fairer (M_diff_ = 3.6, *t*(54) = 18.6, *p* <.001, d = 2.5, 95% C.I. [3.2,4.0]), more enjoyable(M_diff_ = 2.58, *t*(54) = 11.4, *p* <.001, d = 1.53, [2.1,3.0]), more likeable(M_diff_ = 2.1, *t*(54) = 9.5, *p* <.001, d = 1.3, [1.6,2.5]), less annoying(M_diff_ = 3.6, *t*(54) = 16.5, *p* <.001, d = 2.2, [3.1,4.0]), and even more attractive (M_diff_ = .76, *t*(54) = 2.93, *p* <.005, d = .39, [.2,1.3]) than the unfair player. This pattern of results replicates findings of an earlier study using a similar manipulation [2] and shows that fairness manipulated with monetary exchange games is indeed perceived as a socially salient behavior.

### Punishment

Within-subjects t-tests on the punishment behavior demonstrate that participants punished the unfair players significantly more than the fair players (M_diff_ = 3.9, *t*(54) = 7.83, *p* < .001, d = 1.1, [3.0, 4.9]). Moreover, not one participant punished the fair player more than the unfair player. Across participants, punishment of the unfair player emerged in a bimodal distribution (with a peak at zero and a second peak at 10). We used a median split to distinguish between high from low punishers for subsequent analyses. Before applying that split, we subtracted punishment of the fair player (if any) from punishment of the unfair player in order to ensure that we captured fairness-related punishment behavior and not generalized aggression toward the other players.

### Minimum and mean distance

The conventional measure of proxemic behavior is minimum or mean Euclidean distance from the target. We tested for differences between responses to the fair and unfair player in these measures with paired t-tests. In terms of minimum distance, participants came significantly closer to the fair versus unfair target, M_diff_ = -.10 meters, *t*(54) = -2.1, *p* < .05, d = -.30, [-.18,-.01]. This pattern did not emerge for mean distance.

In addition to these within-subject contrasts, we compared the minimum distances of high versus low punishers. Neither mean nor minimum distance was significantly different; there was no significant relationship between traditional measures of proxemics and punishment behavior.

### Proxemic imaging

**Fig 2:**
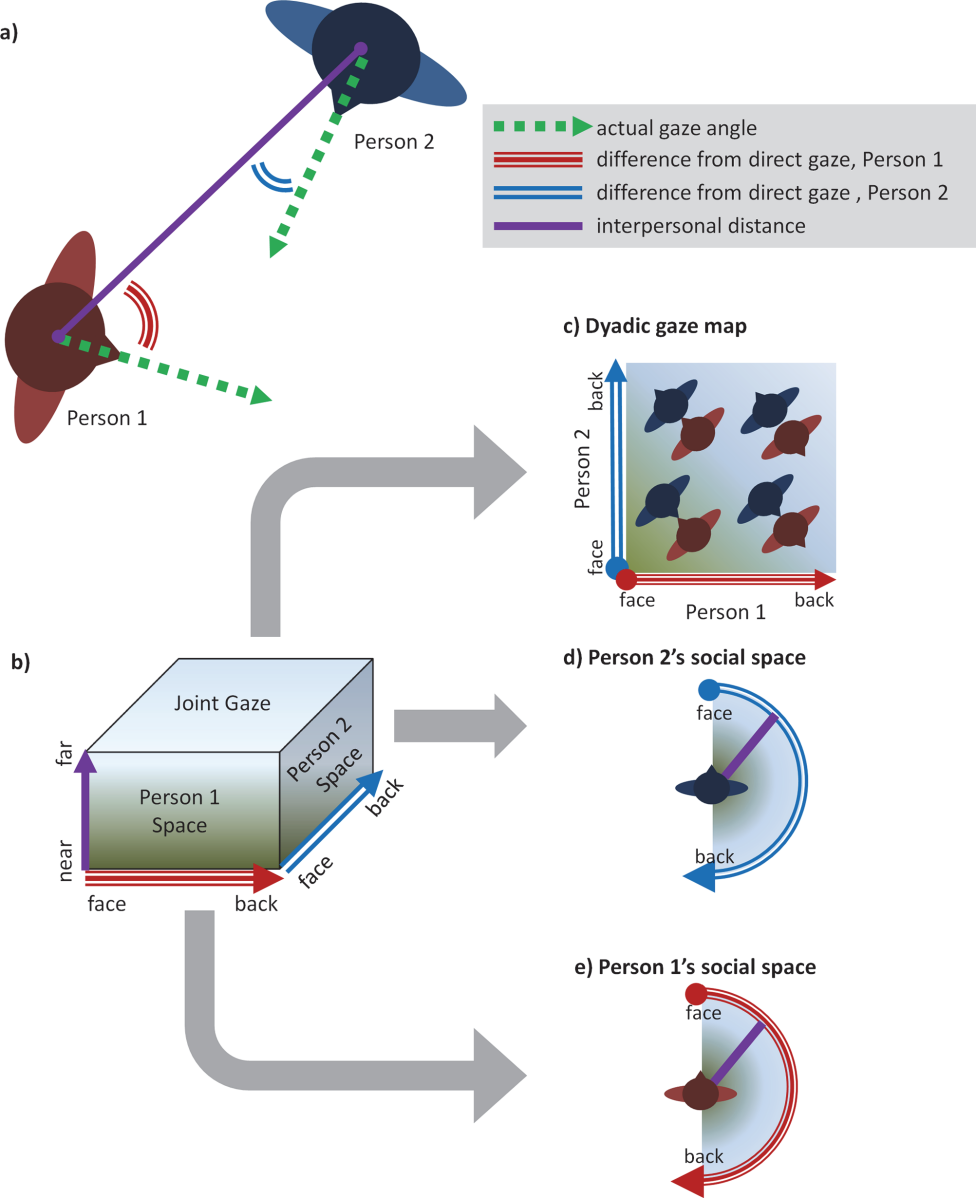
Proxemic imaging. Proxemic images are frequency maps of digital tracking data created using three data points (a): the distance between the two people, and the differences from direct gaze for each person. Each sample of tracking data is binned into a three dimensional space defined by these variables (b). By collapsing across any one of these dimensions, we produce (c) a map of the gaze patters of the dyad (i.e., from face-to-face to back-to-back) as well as (d and e) bird’s eye views of the space around either person.

To compare nonverbal responses to the unfair and fair players in greater detail, we used proxemic imaging (Fig. 2). Proxemic images use position and tracking data from a social interaction between two people to depict where each interactant appeared within the other’s egocentric space, and to depict the joint gaze behavior of the two individuals. To create the proxemic images of an interaction between Person 1 and Person 2, we use three variables for each sample of tracking data: 1) the interpersonal distance between Person 1 and Person 2, 2) the angle at which Person 2 is positioned with respect to Person 1’s head angle, and 3) the angle at which Person 1 is positioned with respect to Person 2’s head angle. Interpersonal distance is simply the Euclidean distance between the two individuals’ heads (in meters). The angle variables are calculated by getting the angular difference between the direction that a person’s head is pointed and the direction that they would point if they were gazing directly at the other person. This value can be anywhere from 0 degrees (when one’s head is pointed directly at the other person), to 180 degrees (when one’s back is to the other person).

For the analysis presented here, the bins were 3 degree x 3 degree x 3.5 centimeters and we binned any tracking samples in which the interpersonal distance fell within 2.1 meters, the range that Hall described as Personal and Close Social space [30]. After binning, the data was then collapsed across each dimension to create three different types of proxemic maps: 1) the egocentric space of Person 1, 2) the egocentric space of Person 2, and 3) a dyadic gaze map. After collapsing, the data were log transformed and smoothed with a Gaussian smoothing kernal (sigma = 7, radius = 21). Using this technique, we created the three proxemic images (Fig. 2C-E) for each of the two dyads of interest: the participant with the fair player and the participant with the unfair player.

In the analyses reported below, we use t-tests to contrast the images on a per pixel basis. To correct for Type II errors as a consequence of multiple comparisons within these contrasts, we used a cluster-based correction via a permutation method [31,32]. Unless otherwise noted, we report clusters that survived a *p* < .05 cutoff.

**Fig 3:**
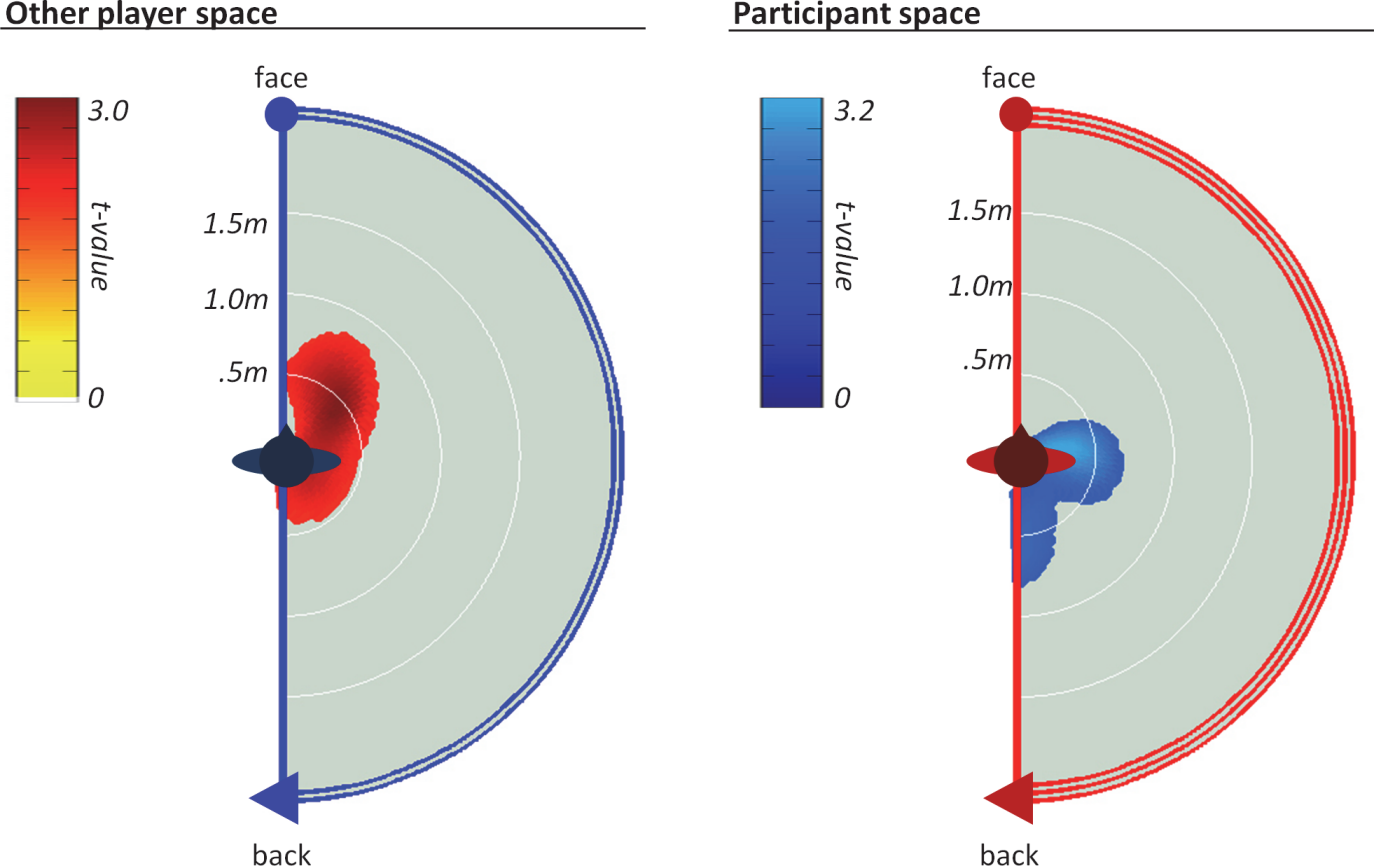
The fairness contrast (fair > unfair). The t-statistic contrasts for the participants’ proxemic responses to fair versus unfair players reveal a significant cluster in the other player’s social space (cluster p <.001) and a marginally significant cluster in the Participant’s social space (cluster p = .07). These patterns reflect relatively more approach of the fair players.

**Fairness versus Unfairness**. To compare proxemic responses to the fair versus unfair players, we used within-subject t-tests for each type of proxemic map (Fig. 3). The contrast of the other players’ space maps reveals that participants were significantly more likely to come close to the fair players (cluster *p* < .001). A similar, although marginal pattern (*p* = .07) emerged in the participant’s space map whereby participants kept the fair player closer to their sides and back (but not front). There were no significant differences in the dyadic gaze maps, suggesting that there were no differences in the joint gaze patterns between the participant and the two types of other players (e.g., no differences in mutual gaze, mutual gaze aversion, or one-sided aversion). Together these contrasts illustrate the predicted pattern whereby participants approached the fair versus the unfair players, although only in terms of interpersonal distance.

**Fig 4:**
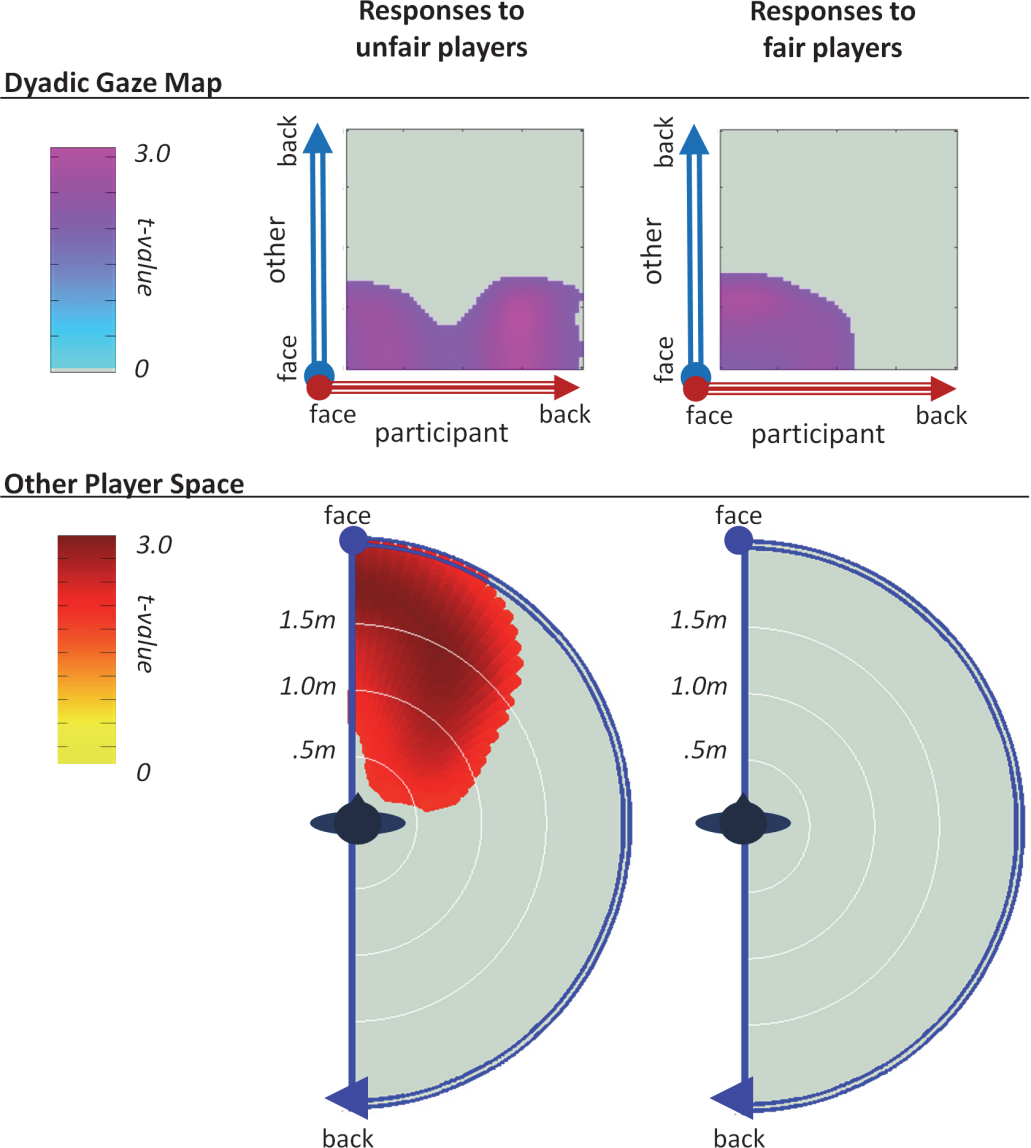
The punishment contrast (high punishers > low punishers). The t-statistic contrasts for the participants’ proxemic responses between high versus low punishers reveal a significant cluster in the unfair player’s space (cluster p <.02) but not the fair player’s space. The dyadic gaze maps further reveal significant clusters for participant responses to both the unfair (cluster p < .01) and fair players (cluster p < .05).

**Individual Differences in Punishment Behavior**. To compare the proxemic responses of the high versus low punishers, we ran an independent samples t-test for each type of proxemic map (Fig. 4). A significant cluster emerged in the unfair player’s space (cluster p<.02) whereby high punishers spent a significantly greater amount of time directly in front of the unfair player than low punishers. They did not, however, spend any more time standing in front of the fair player. The dyadic gaze maps also reveal unique patterns in the high punishers’ treatment of unfair player. Specifically, a significant cluster (cluster p < .01) emerges showing that when high punishers were standing in front of the unfair players, they both looked directly at them and turned their backs to them (cluster p<.01). This was not the case with fair players. Although high punishers were more likely than low punishers to engage in mutual gaze with the fair players (cluster p < .05), they were not more likely to turn their backs on them.

## Discussion

The goal of the present study was to introduce a new method of nonverbal behavior analysis, proxemic imaging, and to test whether it could implicitly detect differential patterns of approach and avoidance behavior during social interactions with fair and unfair individuals. We furthermore sought to test whether proxemic patterns toward the unfair individual would be predictive of subsequent overt behavior, specifically monetary punishment. Given the powerful negative reactions toward fairness violations demonstrated in previous research [2,3], we expected participants to come relatively closer to fair as compared to unfair individuals. However, given that fairness violations can also elicit approach-related responses such as anger and retaliatory behavior [4,5], we also expected that those participants who were more prone to punish unfair others would actually exhibit approach behaviors toward the unfair players.

To test these hypotheses, participants encountered two confederates in a virtual world, one who had played fairly in a sequential iterated Prisoner’s Dilemma and one who had not. In that virtual world, we measured interpersonal distance and gaze between the participants and each of the other players. We then used proxemic imaging to bin these variables into frequency-based maps of each player’s egocentric space and into dyadic gaze maps representing the gaze relationships between the participant and each of the players.

In line with the predictions, these proxemic images revealed that participants came significantly closer to the fair versus unfair players. However, more complex and interesting patterns emerged when we compared the proxemic behavior of participants who were more likely to punish the unfair player in a subsequent task. These individuals spent a significantly greater time in entire area in front of the unfair player. While high punishers engaged in more mutual gaze with both types of players, they also turned their backs to the faces of unfair players.

These patterns suggest that although people tend to implicitly avoid others who have behaved unfairly, the more confrontational among us will place ourselves in full view of unfair others, even if it means turning our backs on them. We believe that these data reflect the mixture of approach and avoidance-related affective responses associated with violations of fairness. On the one hand, the relative avoidance of the unfair players is in line with existing research on proxemics and attitudes; people avoid disliked and approach liked others (e.g. [15,18]). On the other hand, the more direct nonverbal engagement of high punishers with unfair players might reflect approach-related anger and/or aggression [4,5] or, alternatively, a “cooler” motivation to engage with the unfair player in order to enforce social norms [7]. Turning the back may further signal disapproval. Future research might distinguish between the “hot” versus “cold” origins of these responses by including autonomic measurement as indirect indicators of emotional processing.

Another notable pattern in these data is the fact that high punishers spent more time than low punishers engaged in mutual gaze with both players. These data might reflect a greater likelihood of high punishers to observe the others, perhaps to evaluate their intentions or trustworthiness. Future research could explore this question by manipulating the relative ambiguity of a given target’s behavior to test if more ambiguous behavior increases gaze, particularly among participants who are high in the tendency to take action against fairness violations. Such an experiment would help disambiguate between facing another person to aggress versus to understand.

Questions remain regarding the communicative consequences of these types of proxemic patterns. Do the approach and avoidant behaviors observed here convey communicative intentions of our participants such as signaling approval to fair others and censure to unfair others? Or are they merely inadvertent expressions of one’s motivations that are too subtle to be perceived by humans in real time? Further research must address whether or not perceivers are capable of distinguishing between subtle proxemic patterns and determine, in turn, how they affect the target individual and influence the outcome of a social interaction.

Along similar lines, further work could explore the degree to which nonverbal responses to fair versus unfair others are relatively implicit (i.e., automatic and outside of the participants’ awareness) versus more explicit (i.e., conscious and deliberative; [20,22]). Past work demonstrates that people are not necessarily consciously aware of their proxemic responses, and that those responses can even be at odds with explicit self-reports. For example, research has repeatedly shown that subjects who do not explicitly report social prejudices may still exhibit prejudiced proxemic responses, as well as prejudiced responses on other implicit measures [18,19,21]. A similar pattern could be at work here, whereby participants were not consciously aware of their differential nonverbal treatment of the fair versus unfair players. Alternatively, participants’ behavior may have reflected a deliberate and conscious response to fairness violations, particularly on the part of the high punishers who appeared to “face-off” with the unfair players. To address this question, follow-up research could explore the degree to which participants can report their intentions and accurately describe their proxemic behavior when reflecting on the physical qualities of an interaction.

The interaction in this study involved a minimal amount of direct communication between the participant and algorithmically-controlled confederates. These confederates looked at the participant and nodded during the “calibration”, and then stood idling in place for the remainder of the social interaction. Although past research has demonstrated powerful behavioral and physiological effects of the mere presence of avatars in virtual environments (e.g.,[23,33,34]), they are certainly capable of more complex and interactive behaviors than those shown here (e.g., [35,36,37]). Accordingly, future research could explore whether proxemic imaging can capture nonverbal patterns over the course of a more communicatively rich encounter and see if the differential responses towards fair versus unfair individuals change. For example, a similar paradigm could use confederates with a wider behavioral repertoire who interact more directly with participants, either by virtue of using more complex algorithms to drive avatar behavior or by using actual human confederates. Indeed, direct engagement on the part of the confederate via gaze, orienting, or even conversation would likely elicit more direct nonverbal engagement on the part of participants which, in turn, could elicit different patterns of results than those presented here.

Regardless of these open questions, the distinct patterns that emerged in the proxemic images, particularly with regards to high versus low punishers, demonstrate that behavior during a social interaction can involve an interplay of both approach and avoidance. This interplay may be lost when using traditional “one-shot” proxemic measures. Accordingly, although the tendency to come closer to fair than unfair players emerged in both the proxemic images and in one traditional measure (minimum distance), the relationship between proxemics and punishment only emerged in the images. This is true for several reasons. Because the new imaging technique simultaneously illustrates patterns in interpersonal distance and gaze, we can examine the entirety of each interactant’s personal space “bubble”. Because the technique also accounts for both interactants, we can look at coordinated behaviors in gaze direction such as mutual or mutually averted gaze. As such, proxemic imaging provides an account of approach and avoidance at the level of the dyad and treats proxemic outcomes as a jointly determined social phenomenon. Finally, proxemic imaging provides an objective but comprehensive look at an entire interaction, without boiling down the whole time period to one value as with some more traditional measures (i.e., minimum distance).

More broadly, proxemic imaging speaks to an increasing call for studying social cognition in the context of real-time social interactions (e.g., [38,39,40]). The notion here is that by studying actual interactions, we study social phenomena as they naturally occur and not simply as an observer’s responses to socially-oriented stimuli[38]. As such, we are better poised to understand aspects of social cognition that are an emergent property of reciprocal interactions between multiple individuals, aspects that may go unnoticed when we study the individual in isolation [39]. Along these lines, proxemic imaging can provide an objective measurement of an entire dyadic interaction with measures both of the individual (via egocentric maps) and at the level of the dyad (via maps of joint gaze patterns).

Importantly, proxemic imaging could be used to study social interactions between actual humans in the physical world. Although the research reported here used an immersive virtual environment, the method could be employed in any situation in which interactants’ position and orientation are tracked using motion capture technology. As such, the technique could be used to examine actual face-to-face interactions in the laboratory or in “the real world”. Furthermore, by looking at time series of proxemic images, one could study how proxemic behavior unfolds over the course of an interaction or over the development of a relationship. Finally, by gathering the proxemic images of an individual over several interactions with different targets, one could empirically define that individual’s cross-target social space “bubble” [41]. These types of analyses could, in turn, help answer questions regarding the relationships between proxemic behavior and personality, culture, or clinical conditions (e.g., [26,30,42]).
